# Epidemiological and Histopathological Investigation of Sarcoptic Mange in Camels in Egypt

**DOI:** 10.3390/ani10091485

**Published:** 2020-08-24

**Authors:** Marwa A. Ahmed, Ehab Kotb Elmahallawy, Ahmed Gareh, Abdelbaset Eweda Abdelbaset, Fatma A. El-Gohary, Nagwa M. Elhawary, Ahmed K. Dyab, Elzahara Elbaz, Mostafa F. N. Abushahba

**Affiliations:** 1 Department of Pathology, Faculty of Veterinary Medicine, Aswan University, Aswan 24101, Egypt; marwaahmed78@yahoo.com; 2 Department of Biomedical Sciences, University of León (ULE), 24071 León, Spain; 3 Department of Zoonoses, Faculty of Veterinary Medicine, Sohag University, Sohag 82524, Egypt; 4 Department of Parasitology, Faculty of Veterinary Medicine, Aswan University, Aswan 24101, Egypt; ahmedgareh86@gmail.com; 5 National Research Center for Protozoan Diseases, Obihiro University of Agriculture and Veterinary Medicine, Obihiro 080-8555, Japan; abdelbaset2006@hotmail.com; 6 Clinical Laboratory Diagnosis, Department of Animal Medicine, Faculty of Veterinary Medicine, Assiut University, Assiut 71515, Egypt; 7 Department of Hygiene and Zoonoses, Faculty of Veterinary Medicine, Mansoura University, Mansoura 35516, Egypt; dr.fatmagohary@gmail.com; 8 Department of Parasitology, Faculty of Veterinary Medicine, Kafrelsheikh University, Kafr El Sheikh 33511, Egypt; nagwaelhawary@yahoo.com; 9 Department of Parasitology, Faculty of Medicine, Assiut University, Assiut 71515, Egypt; ahmedsaf2001@yahoo.com; 10 Department of Internal Medicine and Infectious Diseases, Faculty of Veterinary Medicine, Mansoura University, Mansoura 35516, Egypt; dr.alzhraa.6712@gmail.com; 11 Department of Medicine, Division of Infectious Diseases, School of Medicine, Washington University, St. Louis, MO 63110, USA; mateya@aun.edu.eg; 12 Department of Zoonosess, Faculty of Veterinary Medicine, Assiut University, Assiut 71515, Egypt

**Keywords:** camel, Egypt, histopathology, prevalence, sarcoptic mange, zoonosis

## Abstract

**Simple Summary:**

Sarcoptic mange is an important zoonotic parasite affecting camel production. Mange zoonosis in camels is complicated by scarcity of available data. One of the main strategies for disease control is early detection of the parasite combined with prevention/control of the major risk factors associated with the infection. The present study focused on the prevalence of sarcoptic mange in camels from Egypt together with a histopathological examination of the parasite and association of the major risk factors, to describe the epidemiological pattern of the disease. Our data demonstrate that 47.6% of the camels harbored sarcoptic mange infections. In addition, the animals exhibited obvious clinical signs of mange and numerous histopathological findings that are consistent with sarcoptic mange. The camel’s age, gender and sampling season were found to be the most significant risk factors associated with the disease. Taken together, our epidemiological and histopathological data are consistent with sarcoptic mange being widespread among camels in the studied area. Our study suggests further research is needed for management of this zoonotic disease in Egypt.

**Abstract:**

Mange has been considered one of the most common parasitic infestations among camels. It adversely impacts animal productivity and poses a risk to human health. Given the scarcity of available data about mange in camels, the current study focused on the prevalence of camel mange and its associated risk factors in Aswan Governorate, Egypt. Towards this end, a general visual inspection was conducted on camels (N = 210) in different markets and slaughterhouses in Aswan Governorate. Skin scrapings from suspect infected camels were also examined microscopically. Importantly, these findings were further checked and confirmed by histopathology on samples from suspected cases collected post-slaughter in abattoirs. The possible risk-associated factors, which include the camel’s age, sex and sampling season, were recorded and statistically analyzed. Interestingly, the data showed that a total of 100 camels (47.6%) were found exclusively infested by sarcoptic mange. Furthermore, the predominant histopathological changes included burrowing tunnel of mites in the skin, hyperkeratosis and acanthosisconsis of the epidermis, while the dermis showed hemorrhage, mononuclear inflammatory cell infiltration around the blood vessels and perifolliculitis. These major histopathological findings are consistent with sarcoptic mange. Furthermore, the statistical analysis of the possible associated risk factors, camel’s age (*p* = 0.006), gender (*p* = 0.032) and sampling season (*p* = 0.004), were all found to be significantly affected and related to the disease. In this regard, camels ≥2 years old were found at higher risk of infection (odds ratio (OR) = 2.75; 95% confidence interval (CI), 1.345 to 5.604) versus younger animals (OR = 0.36; 95 CI, 0.1784 to 0.743). Females had higher odds of exposure (OR = 2.02; 95% CI, 1.096 to 3.708) compared to males (OR = 0.50; 95% CI, 0.269 to 0.912). Moreover, the exposure to infection was reported higher in winter (OR = 2.30; 95% CI, 1.297 to 4.098) than in summer (OR = 0.43; 95% CI, 0.244 to 0.771). Collectively, our data provide novel epidemiological and histopathological support for sarcoptic mange being widespread among camels in the studied area. Sarcoptic mange is extremely contagious and zoonotic. Therefore, our baseline investigation indicates an urgent need for additional multicenter-studies to investigate the occurrence of this disease in camels and humans combined with the appropriate control measures of camel importation for combating this disease.

## 1. Introduction

The one-humped camel (*Camelus dromedarius*) or Arabian camel possesses a myriad of unique adaptive physiological and anatomical traits, making them an important component of the desert and non-desert ecosystems [1,2,3]. In addition, the use of camels in work and as a source of milk and meat has been drastically increased by Egyptians in the recent years [4]. In Egypt, camel production is practiced on a small scale and the country demands are met by importation, particularly from Sudan [5]. However, the economic impact of camel production is hampered by highly contagious and zoonotic diseases, inappropriate veterinary services and feed insufficiency [6,7]. Of particular concern, mange was found to be the second most common parasitic disease infesting camels, preceded only by trypanosomiasis, and its zoonotic potential has been documented in several studies [8,9,10,11,12,13]. Clearly, mange is considered a substantial infectious and debilitating skin disease affecting camels [13,14,15]. Importantly, sarcoptic mange is considered the most common identified mange in camels, while chorioptic mange is rare [13,14]. The causative agent of sarcoptic mange in camels is *Sarcoptes scabiei* var. *cameli*, which is a tiny rounded parasite, whereas the dimensions of the female and male is 330 to 600 µm × 250 to 400 µm and 200 to 240 µm × 150 to 200 µm, respectively [16,17]. Mange is tenacious and not susceptible to common chemicals, making eradication quite difficult [18]. Besides the contagious nature of sarcoptic mange, it is also significant from a zoonotic point of view [15,19,20]. Humans contract the infection through direct contact with infected animals while inter-animal transmission occurs through direct contact or via infected fomites, such as trees, rugs and luggage [21,22].

It should be stressed that sarcoptic mange in camels is recurrent, abrupt in onset and repeatedly starts on the medial parts of the thigh or inguinal area, the neck or the flanks [23]. Its incidence is regularly connected with hygienic practices and nutrition and, therefore, high infection risk has been recorded in in camels with reduced hygiene management or malnutrition [24]. In affected animals, the lesions gradually progress from pruritic nodules that lead to scratching and eventually render the skin hairless, crusty and wrinkled, especially on the thighs, hocks and axillae [25]. The clinical picture of sarcoptic mange in camels includes intense pruritus, exudative dermatitis, parakeratotic scaly crust formation, alopecia, and dark, thickened skin [24]. Fissures develop in the crust and underlying epidermis, resulting in hemorrhages. Emaciation, debilitation, anemia and subcutaneous edema are common signs of mange in camels [26]. During the development of mange, itchiness distracts the animals from eating so that they often become emaciated [26,27]. Given the fact that the Aswan Governorate is considered a primary station for imported camels in Egypt, as well as the veterinary and public health significance of camel mange, the current study was undertaken to explore the prevalence of the disease in camels and the associated risk factors. The present study represents an epidemiological overview about mange in camels in Egypt and provides novel data on this topic. The present work also involved studying the histopathological changes in camel skin due to sarcoptic mange as a confirmatory tool for verification of the parasitological and epidemiological findings.

## 2. Material and Methods

### 2.1. Ethical Considerations

The study protocol was carefully reviewed and approved by the local guidance body on Research, Publication and Ethics of the Faculty of Veterinary Medicine, Mansoura University, Egypt, which complies with all the relevant Egyptian laws.

### 2.2. Study Area and Animal Data

The present study was conducted on camels showing skin lesions in different regions in the body. Those animals were from different geographical areas (Idfu, Kawm-Umbo and Abu-Simbel) in Aswan Governorate, Egypt, between June 2019 and March 2020. Aswan Governorate is located in the southernmost part of Upper Egypt located close to the Sudanese border. Imported Sudanese camels are normally quarantined for 10–15 days in the Abu Simbel or Shelateen facilities in Aswan before their release to different markets and slaughterhouses. A total of 210 dromedary camels of different age and sex (60 females and 150 males) were recruited in this current study. These camels were primarily imported from Sudan for the purposes of work, breeding and/or slaughtering. Sampling was conducted during different seasons to record the seasonal variations of the disease in camels.

### 2.3. Collection of Skin Scrapings

Following a general visual inspection of the animals, the suspected camels with skin lesions were clinically scored according to a system previously applied to horses with chorioptic mange [28], as follows: 0: no clinical signs; 1: mild signs; 2: moderate signs; and 3: severe signs. For sample collection, suspected camels with cutaneous lesions were restrained properly and the hairs were clipped from the margins of the lesions with the help of scissors. After sterilization of the skin, profound skin scraping was completed in different body areas, including the head, neck, flanks, front and posterior limbs of the affected animals, with the aid of a scalpel blade [21]. In the slaughterhouses, deep skin scrapings from the ends of the examined lacerations of the slaughtered camels were collected in petri plates and transported to the parasitology laboratory at Assiut University for further processing and examination, which included additional parasitological and histopathological investigations [29].

### 2.4. Clincal Manfestations

The lesions observed on the examined camels were recorded and ranged from mild to severe and scored on a scale from 1 to 3. Skin samples from animals showing mild clinical signs were given a score of 1, which include localized alopecia, crusting, scaling or lichenification, thickening and corrugation of skin, scab formation, pruritic dermatitis and intense itching. In turn, moderate symptoms (a score of 2) were represented by extensive alopecia, crusting, scaling or lichenification with or without mild excoriation. On the other hand, severe signs (a score of 3) were observed as severe excoriations in addition to other signs, such as debilitation, anemia and subcutaneous edema.

### 2.5. Parasitological Examination

The parasitological examination of mange was done using a previously published protocol [30]. Briefly, the collected skin scraping was mixed with a small amount of 10% potassium hydroxide solution and heated till just boiling or left to stand for 0.5–1 h until the skin particles have partly disintegrated. Briefly, skin scrapings from the edges of the clinical lesions were collected in labeled Petri dishes, the edges of which were smeared with Vaseline so as to prevent the mites from escaping. The collected skin scrapings were transferred to test tubes and mixed with a small amount of 10% potassium hydroxide solution and heated till just boiling or left to stand for 0.5–1 h until the skin particles have partly disintegrated. The tubes were centrifuged at 3000 revolutions per minute. The supernatant fluid was discarded, and a drop of sediment was investigated under a stereoscopic microscope (Olympus, Life Science Solutions, Tokyo, Japan) for the detection of the various stages of sarcoptic mites and their eggs.

### 2.6. Histopathological Examination

Skin samples from different areas of the body with lesions (neck, forelegs, hind legs, back, flank and abdomen) were also collected from Dromedary camels (N = 20) slaughtered in abattoirs with suspicion of mange for diagnostic purposes. As shown in Table 1, out of those twenty samples randomly collected for the histopathological examination, four animals showed mild clinical signs and six camels showed moderate clinical signs; the remaining samples had severe clinical signs (N = 10). These randomly selected samples were fixed in 10% neutral buffered formaldehyde. Samples were then processed by sectioning where the paraffin-embedded tissues were sectioned to a 4–5 µm thickness. Sections were mounted on slides, stained by hematoxylin and eosin (H&E) and then examined under light microscopy for the presence of mange mites and any associated histopathological changes [31,32]. The resulting histopathological changes were then ranked according to their degree of severity into three categories: mild, moderate and severe histopathological findings, as described elsewhere [33].

### 2.7. Statistical Analysis

Fisher’s exact tests and odds ratios with 95% confidence intervals (95% CI) were used in our study to analyze the impact of the different variables (age, sex and season) on the disease prevalence in camels. GraphPad Prism software (JMP, Cary, NC, USA) was used to compute the data. A *p* value < 0.05 was considered significant.

## 3. Results

### 3.1. Epidemological Data and Clinical Mainfestations

The present study showed that all camels are remarkably exposed to mange caused by sarcoptic mites. The prevalence of mange in the population of this study was 47.6% (100/210). The highest prevalence of mange was reported among animals of more than two years of age (52.7%). Furthermore, a higher infection rate (60%) was recorded in female camels compared to males. Additionally, the highest prevalence of mange was detected in the winter season (55.4%). Analysis of the results revealed statistically significant differences related to the age, sex and season categories of the studied camels (*p* < 0.05), which are shown in Table 2.

**Figure 1:**
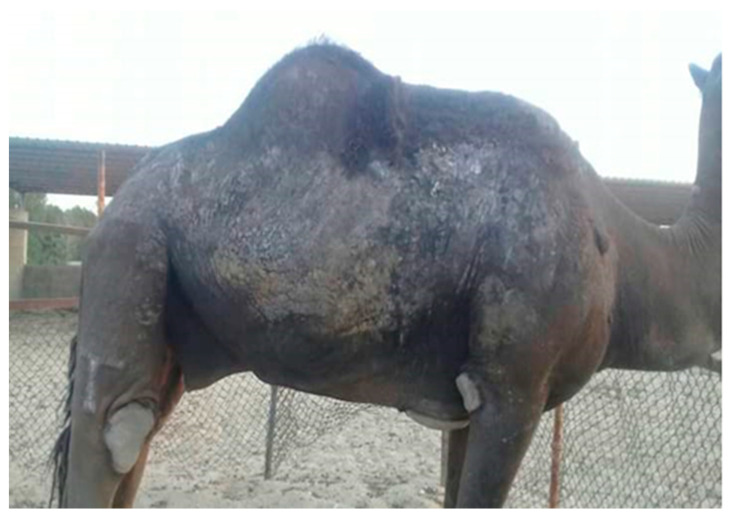
A camel showing some clinical manifestations of sarcoptic mange that includes multiple clinical signs, including thickening and corrugation of the skin, scab formation, pruritic dermatitis and hair loss.

In accordance with the clinical manifestations, the infected animals showed various clinical signs, which are reported in Figure 1. The skin of some affected camels often became discolored to a slate grey color. These lesions were distributed in various areas of the body, particularly the areas with thin skin, including the neck, abdomen, prepuce and flank. As result of the infection, some animals were emaciated and exhibited erythema and numerous small vesicles combined with itching and then scratching against hard objects, such as walls, leading to redness and scab formation in the affected areas. Out of 100 positive skin scrapings, 15 skin samples originated from animals showing mild clinical signs (a score of 1), 40 from animals with moderate signs and 45 from animals with severe signs.

### 3.2. Parasitological Examination, Mange Prevalence and Risk Factors

**Figure 2:**
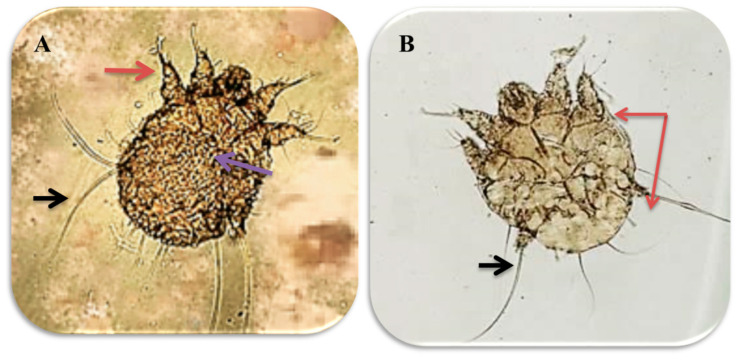
Male and female sarcoptic mange mites under 10× magnification: (**A**) *Sarcoptic scabiei* female, with transverse ridges and triangular scales on the dorsum (pink arrow). The 1st and 2nd pairs of legs ended with cup-shaped suckers (red arrow) while the 3rd and 4th ones ended with bristles (black arrow); (**B**) *Sarcoptic scabiei* male, with the 1st, 2nd and 4th pairs of legs ending with cup-shaped suckers (red arrow) and the 3rd pair ended with bristles (black arrow).

The parasitological examination revealed the presence of male and female mites, which are shown in Figure 2.

The prevalence of sarcoptic mange in the examined camels was 47.6% (100 out of 210). The mange mite infestation in dromedary camels was significantly affected by the animal’s age, sex and season. Camels of age two or older were found to be at higher risk (OR = 2.75; 95% CI, 1.345 to 5.604) than those younger than 2 years (OR = 0.36; 95 CI, 0.1784 to 0.7435). Regarding gender, females were more affected than males with an odds ratio of exposure equal to 2.02 (95% CI, 1.096 to 3.708) and 0.50 (95% CI, 0.2697 to 0.9127), respectively. Moreover, a seasonal variability of the disease was observed and animals were found to be at higher risk in winter than in summer (Table 2).

### 3.3. Histopathological Findings

**Figure 3:**
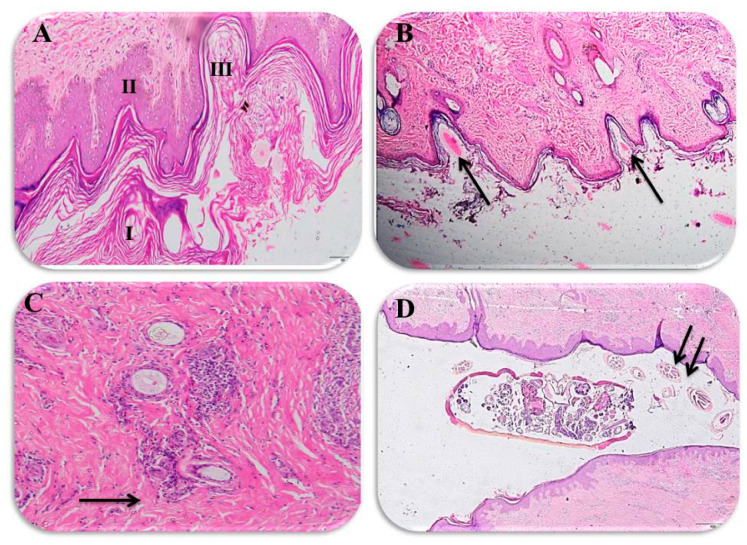
Histopathological findings for the infected camels: (**A**) the epidermis is thickened by compact hyperkeratosis (I) and acanthosis (II), which are in response to both the mite itself and also to the self-trauma caused by intense pruritus, and also shows the burrowing tunnels of the mites (III), ×100 magnification; (**B**) histological section showing *Sarcoptic scabiei* eggs in tunnels in the epidermis of an infested camel (arrow), ×200; (**C**) dermis showing inflammatory cell infiltration around the blood vessels and perifolliculitis (arrow), ×100; and (**D**) adult *Sarcoptic scabiei* and its eggs within the migrating tunnels of the camel skin (arrow), ×200.

Interestingly, our present data reveals major histopathological findings that are consistent with sarcoptic mange. The microscopical examination of the affected animals demonstrated histopathological changes in the epidermis and dermis. As previously mentioned, these changes were scored into mild (a score of 1), moderate (a score of 2) and severe (a score of 3). In this regard, the collected samples from animals with severe clinical signs exhibited a score of 3 and were histopathologically represented by thickness in the epidermis, dense hyperkeratosis and acanthosis (Figure 3A,B). Meanwhile, Figure 3C depicts that skin of a camel exposed to the burrowing tunnels of the mites, inflammation and hemorrhage in the dermis, and these samples represent a score of 2 for those animals that showed moderate clinical signs. On the other hand, camels with mild clinical signs showed inflammatory cell infiltration on the dermis near the blood vessels and perifolliculitis that was classified as a score of 1 (Figure 3D). Taken together, the histopathology scoring seems to be consistent with the clinical signs that are shown in Table 1.

## 4. Discussion

Despite its veterinary and zoonotic burden, camel mange research is minimal at the national and international level. Clearly, there is an obvious scarcity in the available data about the disease in Egypt, with the exception of one previous study in upper Egypt [29], making it difficult to set out the appropriate mange control measures. To the authors’ knowledge, camels can be infected by sarcoptic and chorioptic mange mites [24]. However, it should be understood that sarcoptic mange, caused mainly by *Sarcoptes scabiei* var. *cameli*, is considered the most common type of mange in camels while chorioptic mange is relatively rare worldwide [13,14]. Besides being the most common and reported, sarcoptic mange has been considered an extremely contagious and serious zoonosis in camels [15,34]. Our work provides novel interesting data related to the high occurrence of sarcoptic mange in Egypt. Furthermore, our study investigated the major associated risk factors with the occurrence of mange combined with very interesting histopathological changes of the disease among the studied animals. The southernmost Aswan Governorate is considered the primary station of imported camels, which from there are further distributed to the entire country.

As shown in our results, the prevalence of mange mite infestation in the examined camels was 47.6%. Our study reports a higher prevalence than a previous study in Upper Egypt, which reported a mange prevalence of 6.06% among 660 examined one-humped camels [29]. Moreover, a lower prevalence rate of 25% was recorded among camels in Sudan [35]. In Ethiopia, prevalence rates of 16.7% and 35.4% were documented [23,36]. Furthermore, lower prevalence rates of 11.28% and 42.22% were reported for camel mange in Pakistan [21,37]. On the other hand, our results are slightly lower than a previous study in Sudan where a prevalence rate of 55.2% was recorded for mange among camels [38]. The variation in the prevalence rate of our present study versus previous studies seems to be multifactorial in origin. The potential factors that could be attributed to the contagiousness of mange, besides other factors, are bad management practices in animal premises, inadequate veterinary care, and a lack of awareness among camel owners, which might also have contributed to the high infestation rate among the examined camels [7,39]. Tracking of the precise origin of the disease remains difficult due to the long incubation period of camel mange (2–3 weeks), and the imported Sudanese camels are quarantined in the Abu Simbel or Shelateen facilities only for 10–15 days before release to markets and slaughterhouses [40].

Among the analyzed variables in this study, age was found to be a significant factor affecting mange infestation in camels. Animals of more two years of age had a greater odds ratio of exposure than those of younger age. Similarly, older camels were found more susceptible to infection compared to younger ones in previous studies [21,36]. A previous study in sheep reported a higher prevalence of mange infestation in adult sheep compared to young ones [41]. However, a previous study reported an opposite conclusion related to the higher occurrence of the disease in younger camels than older ones [29,36]. Furthermore, our present results are not consistent with previous reports in domestic animals, such as cattle, and wild animals, such as Iberian wolves and cheetahs, that reported a higher prevalence in young animals [42]. Meanwhile, another previous study has stated that both very young and aged camels are mostly liable [43]. It is noteworthy to state the fact that young camels are usually kept indoors under intensive rearing while older ones participate in work and grazing that might justify the higher susceptibility of older animals than younger ones [35].

Gender was another significant risk factor in our present investigation, whereas females were at higher risk of being infested by mange compared to males. Our finding is consistent with a previous study in Pakistan [37]. Furthermore, the present results are similar to previous studies carried out in domestic and wild animals from Kenya [42]. It seems that the higher level of prolactin and progesterone hormones enhances the vulnerability of the females to any infection. Furthermore, pregnancy, lactation and the breeding behavior of mange-infected males may aggravate the problem and could be an additional risk factor [37]. In stark contrast, other previous reports recorded a higher prevalence of sarcoptic mange in males than females, as was reported in Egypt, Ethiopia and Sudan [29,35,36]. However, a previously published report detected a similar infestation rate of mange in male and female sheep [41]. Several studies documented the impact of seasonality on camel mange [12,23,24,29,37,43]. Among others, one study demonstrated that camel mange is more prevalent in summer than winter [43], while the remaining studies reported the opposite, a higher prevalence of mange in winter than summer, which is consistent with our present findings [21,23,29,37]. The explanation behind the seasonal variability was reported in the latter studies, where low temperatures combined with the overcrowding during the winter months were reported to provide favorable conditions for rapid propagation of the mite life cycle as well as easy spread between susceptible animals [44,45].

To the authors’ knowledge, a histopathological examination of tissue biopsies is an important diagnostic tool for detection of various infectious agents in situ [46,47]. In our study, infection by camel mange was associated with certain histopathological changes that were scored into mild, moderate and severe degrees. Importantly, those histopathological changes were in the form of dermal hyperkeratosis (orthokeratosis and parakeratosis), epidermal hyperplasia, epidermal tunnels, creation of a crust and dermatitis, which is consistent with several previous reports [48]. Furthermore, the reported histopathological features in the current study are in agreement with previous studies regarding mange in various animal species [49,50]. Despite this consistency, discrepancies in severity and dispersal of the skin lesions were encountered even with animals of the same species, possibly due to the variation in immune responses between different animals [33]. It should be stressed that the histopathological changes triggered by mange mites in the examined camels are largely due to the parasite’s burrowing behavior as well as the defensive response from the affected hosts [51]. Sarcoptic mange is widely known as burrowing mites that make tunnels in the skin of the infested host where they lay eggs and continue their lifecycle [52,53]. Along this course, an enormous aggregate of antigenic material is released in the skin, including dead mites, sloughed skin of the living adult and immature mites, and eggshells, leading to an increase the hypersensitivity to the mites [53,54]. Moreover, excavations on the skin, particularly those made by mature females, resulted in induction of thickening of the epidermis and crust formation [55], and these observations are consistent with our present findings. It is noteworthy to mention that the histopathologic findings in the present study were consistent with the macroscopic lesions, which is in agreement with a previously published report [33].

## 5. Conclusions

Given the above information, the present study demonstrates a high occurrence of sarcoptic mange infestation among camels in Aswan, Egypt, which is supported by the interesting histopathological data. Though underestimated, this could be associated with loss of productivity among the affected individuals and a zoonotic risk for camel traders, camel breeders and slaughterhouse workers. Clearly, it is of utmost importance to undergo periodical studies to track its prevalence in camels and contact people. The role of veterinary services is also important to advise farmers about the zoonotic importance of disease and to maintain their animals free from ectoparasites by keeping the animals under hygienic conditions. Further future investigation and a wide surveillance strategy seems warranted to explore this parasitic zoonosis in the entire country, which is crucial for combating this disease of public health concern.

## Figures and Tables

**Table 1:** Macroscopic and microscopic scoring of infected camels.

Clinical Signs Score	Number of Infected Animals	Selective Samples for Histopathology	Score of Histology		
			Mild Lesions (1)	Moderate Lesions (2)	Sever Lesions (3)
Mild (1)	15	4	3	1	0
Moderate (2)	40	6	0	4	2
Severe (3)	45	10	0	2	8

**Table 2:** Prevalence of camel mange adjusted for age, sex and season.

Variable		No. Examined	Positive No. (%)	Negative No. (%)	Odds Ratio (95% CI), *p* Value
Age	≤2 year	45	13 (29)	32 (71)	2.75 (1.34 to 5.60) *p* = 0.006
	>2 year	165	87 (52.7)	78 (47.3)	
Sex	Female	60	36 (60)	24 (40)	2.02 (1.10 to 3.71) *p* = 0.032
	Male	150	64 (42.7)	86 (57.3)	
Season	Summer	80	28 (35)	52 (65)	2.30 (1.30 to 4.10) *p* = 0.0045
	Winter	130	72 (55.4)	58 (44.6)	
Total		210	100 (47.6)	110 (52.4)	
